# Editorial: Immune response to SARS-CoV-2 and implications for clinical outcome

**DOI:** 10.3389/fimmu.2023.1233291

**Published:** 2023-07-10

**Authors:** Edwin Bölke, Martijn van Griensven, E. Marion Schneider, Johannes C. Fischer, Torsten Feldt, Verena Keitel, Wilfried Budach, Jan Haussmann, Danny Jazmati, Christiane Matuschek

**Affiliations:** ^1^ Department of Radiation Oncology, University of Dusseldorf, Dusseldorf, Germany; ^2^ Department cBITE, Institute for Technology-Inspired Regenerative Medicine, Martijn van Griensven Maastricht University, MERLN, Maastricht, Netherlands; ^3^ Division of Experimental Anesthesiology, University Hospital Ulm, Ulm, Germany; ^4^ Institute for Transplantation Diagnostics and Cell Therapeutics, Medical Faculty, Heinrich-Heine-University, Dusseldorf, Germany; ^5^ Clinic of Gastroenterology, Hepatology and Infectious Diseases University Hospital Dusseldorf, Medical Faculty, Heinrich-Heine-University Dusseldorf, Dusseldorf, Germany

**Keywords:** COVID - 19, cytokine, sepsis, inflammation, pandemic

Since the outbreak of the SARS-CoV-2 pandemic in 2019, our understanding of the disease and its pathophysiology has evolved, paving the way to more effective therapeutic and prophylactic approaches (Krysko et al.). As a result of the global vaccination campaign, improved management of severe COVID-19 patients, but also due to decreased pathogenicity of the currently prevailing SARS-CoV-2 variants, mortality and hospitalization due to COVID-19 have been significantly reduced, especially in developed countries increasing natural immunity. Since recently, a major view addresses organ dysfunction in COVID-19 patients leading to improved outcome (1) Nevertheless, the precise nature of an efficient immune response to SARS-CoV-2 remains ill-defined. Inflammation and immune activation are essential but hyperinflammation may turn into a detrimental condition (Krysko et al.). As a consequence, the immune response to SARS-CoV-2 is responsible for a significant proportion of multiple faces of the pathogenicity spectrum (Krysko et al.). SARS-CoV-2 is continuously mutating and poses a major challenge to cellular and humoral immunity, as well as vaccination. On the other hand. The goal of this Research Topic was to present new data regarding the immune response to SARS-CoV-2 and its implication for the clinical outcome.

Saleki et al. showed in this Research Topic that Fas/FasL pathways could play a major role in the mediation of hyperinflammatory cytokine response in COVID-19. According to their results, Fas/FasL pathways are consumed through an interaction with MMPs, and the novel triangle of viral entry, cytokine storm, and multi-system injury. They detected in their investigation that the role of Fas/FasL may depend on the stage of the disease and the severity of COVID-19. By in silico analysis they showed that alterations in the blood of COVID-19 patients influence Fas/FasL interactions at the molecular level. Preclinical and clinical research by antibodies directed against sFAS for COVID-19 treatment could be an option attenuate COVID-19 pandemic severity.

Daamen et al. detected unique transcriptional signatures as surrogate parameters for disease severity. They found immune profiles of severe COVID-19 patients to be of prognostic value for the outcome of the infection. Such alterations in COVID patients could be engineered to better allocate healthcare resources and help to develop targeted treatment plans for i) better care and ii) to combat the worse outcomes.

Lapointe et al. fully characterized humoral responses in a laboratory-confirmed case of serial infection by SARS-CoV-2 Omicron subvariants BA.1 and BA.2 in a patient who showed typical immune responses to three vaccination doses of a COVID-19 mRNA vaccine. Whereas data on repeat Omicron infections are still limited, a recent genomics-based trial from Denmark described 47 cases of BA.2 reinfection that happened between 20 and 60 days following BA.1 infection (24). The authors hypothesize that such events were rare (<0.1% of cases during the brief window of analysis) and would more likely happen among unvaccinated individuals, but additional evaluation of the data shows that most reinfection cases were due to BA.2 following BA.1.

Halliday et al. presented in their trial that low-volume in-house antibody assays are of sufficient diagnostic value. Their study demonstrates the importance of using well-characterized samples and controls for all stages of assay development and evaluation. These cost-effective assays may turn out to be predominantly valuable for seroprevalence studies in developing countries.

Krysko et al. indicated that the severity of SARS-CoV-2 infection correlates with high numbers of alveolar mast cells and their degranulation. Accordingly, SARS-CoV-2 correlated with activation of mast cells, significantly. Mast cell activation appears to be due to indirect effects related to the overall inflammation. Their data recommend that proteases from mast cells might forecast the clinical outcome and sequelae of severe COVID-19 infections. As a consequence, patients might profit from including mast cell stabilizing drugs in their novel treatment scheme.

Penrice-Randal et al. suggest that blood gene expression profiles predict intensive care unit admission in hospitalized patients with COVID-19. Their team demonstrated a “best gene” with its expression signature would be predictive for ICU admission. For this aim, they applied topological data analysis with an accuracy of 0.72 (ROC AUC: 0.76). The gene signature describes herein targeted differentially activated pathways controlling epidermal growth factor receptor (EGFR) presentation, Peroxisome proliferator-activated receptor alpha (PPAR-α) signaling and Transforming growth factor beta (TGF-β) signaling.

Rubio et al. demonstrated that SARS-CoV-2 infection during the third trimester of pregnancy leads to a robust antibody and cytokine response at delivery and causes a significant reduction of the SARS-CoV-2-specific IgGs transplacental transfer, with a significant negative effect when the infection is closer to delivery.

Bizjak et al. emphasized the biomarker, Kynurenine in serum and saliva. Kynurenine may play a role in acute and long-term pathophysiology of the SARS-CoV-2 disease. Specifically, Kynurenine is induced by interferon-g (IFN-g) by innate immune cells (NK-effectors), but also by TH17/IFN-g inflammatory T cells (ref.). The most prevalent pathway of induction in both Long- and Post-COVID for diagnostics and syndrome monitoring needs to be addressed in further studies.

Castelli et al. from Brazil assumed that MUC22, (a member of the mucins’ family), may play an important protective part against severe Covid-19. They think that MUC22 might reduce the overactive immune responses in the senior population. Interestingly, the resilient super elderly group showed a higher frequency of some missense variants in the MUC22 gene as one of the most robust signaling elements in the MHC region. These results are based on comparison of the severe Covid-19 group to a general elderly control population.

Schenz et al. from Germany showed an elevated occurrence of clonal hematopoiesis of indeterminate potential in hospitalized patients with COVID-19. Their results indicate an increased exposure to a severe course of COVID-19 requiring hospitalization related with CHIP. Furthermore, they relate it to a differentially regulated cellular immune reply under the pressure of SARS-CoV-2 infection.

Therefore, a better understanding of the immunological background of the disease is of utmost importance in order to develop more effective prevention and treatment strategies.

## Author contributions

EB, MvG, EMS, JCF, TF, WB, JH, DJ, VK, CM contributed to drafting and writing the article. All authors contributed to the article and approved the submitted version.

## References

[1] Borlotti A Thomaides-Brears H Georgiopoulos G Banerjee R Robson MD Fusco DN. The additive value of cardiovascular magnetic resonance in convalescent COVID-19 patients. Front Cardiovasc Med (2022) 9. doi: 10.3389/fcvm.2022.854750 PMC9021393 35463767

